# Long-Reads-Based Metagenomics in Clinical Diagnosis With a Special Focus on Fungal Infections

**DOI:** 10.3389/fmicb.2021.708550

**Published:** 2022-01-06

**Authors:** Minh Thuy Vi Hoang, Laszlo Irinyi, Yiheng Hu, Benjamin Schwessinger, Wieland Meyer

**Affiliations:** ^1^Molecular Mycology Research Laboratory, Centre for Infectious Diseases and Microbiology, Faculty of Medicine and Health, Sydney Medical School, Westmead Clinical School, The University of Sydney, Sydney, NSW, Australia; ^2^Westmead Institute for Medical Research, Westmead, NSW, Australia; ^3^Sydney Infectious Disease Institute, The University of Sydney, Sydney, NSW, Australia; ^4^Research School of Biology, Australia National University, Canberra, ACT, Australia; ^5^Westmead Hospital (Research and Education Network), Westmead, NSW, Australia

**Keywords:** pathogenic fungi, long-read sequencing, metagenomics, identification, diagnosis, mycoses

## Abstract

Identification of the causative infectious agent is essential in the management of infectious diseases, with the ideal diagnostic method being rapid, accurate, and informative, while remaining cost-effective. Traditional diagnostic techniques rely on culturing and cell propagation to isolate and identify the causative pathogen. These techniques are limited by the ability and the time required to grow or propagate an agent *in vitro* and the facts that identification based on morphological traits are non-specific, insensitive, and reliant on technical expertise. The evolution of next-generation sequencing has revolutionized genomic studies to generate more data at a cheaper cost. These are divided into short- and long-read sequencing technologies, depending on the length of reads generated during sequencing runs. Long-read sequencing also called third-generation sequencing emerged commercially through the instruments released by Pacific Biosciences and Oxford Nanopore Technologies, although relying on different sequencing chemistries, with the first one being more accurate both platforms can generate ultra-long sequence reads. Long-read sequencing is capable of entirely spanning previously established genomic identification regions or potentially small whole genomes, drastically improving the accuracy of the identification of pathogens directly from clinical samples. Long-read sequencing may also provide additional important clinical information, such as antimicrobial resistance profiles and epidemiological data from a single sequencing run. While initial applications of long-read sequencing in clinical diagnosis showed that it could be a promising diagnostic technique, it also has highlighted the need for further optimization. In this review, we show the potential long-read sequencing has in clinical diagnosis of fungal infections and discuss the pros and cons of its implementation.

## Introduction

Rapid and accurate diagnosis of pathogens is essential in the management of infectious disease and is the ultimate goal for clinical microbiology laboratories. Successful diagnosis ensures effective treatment, with patient outcome improved the faster the pathogen is identified (Perfect, 2013). The ideal diagnostic test would be capable of reliably identifying any potential pathogen, provide additional information, such as the antimicrobial resistance profile, and offer the potential for further epidemiological analysis, while remaining cost-effective (Wickes and Wiederhold, 2018). The current available identification techniques for fungal pathogens are unable to meet all these criteria. The main drawback of the wide range of traditional identification techniques is that they rely in general on the *in vitro* growth of the causative pathogen before its diagnosis (Irinyi et al., 2016).

Sequence-based metagenomics is a powerful culture independent tool for the identification of mixed microbial communities, regardless of the ability of member organisms to be grown *in vitro* (Miller and Chiu, 2018). Recently, developed long-read sequencing technologies generate sequencing reads far longer than previous sequencing technologies and are capable of addressing the shortcomings of conventional identification, inaccurate, time consuming, requires specific expertise, and short-read based metagenomics, which is unable to detect all pathogens when based on short DNA fragments (Tedersoo et al., 2021).

*This review explores previous and potential use of long-read sequencing in the clinical diagnosis, focusing on fungal infections.*

## Non-Sequencing-Based Identification Techniques

**Figure 1:**
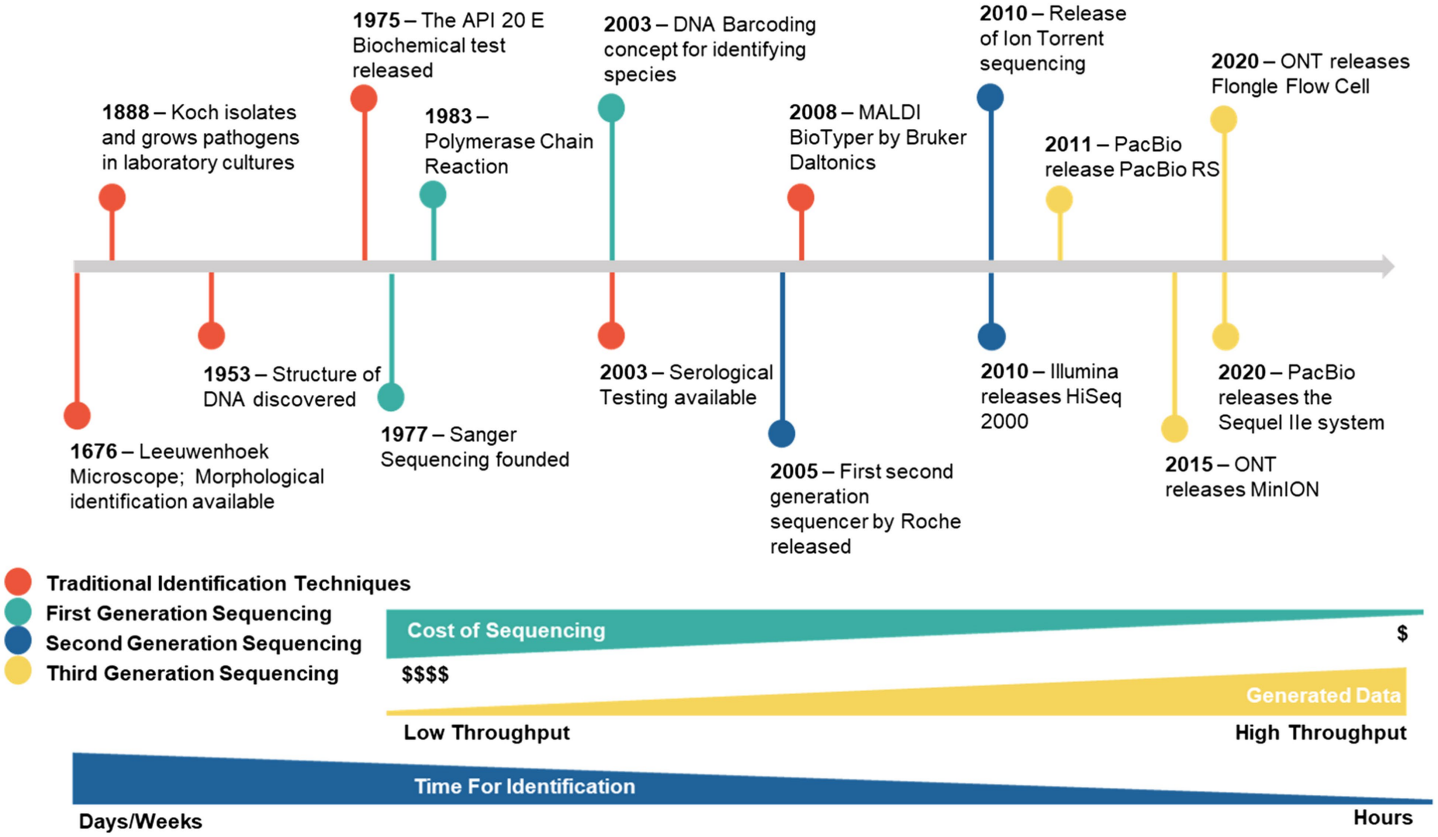
History of different identification techniques applied for the diagnoses of fungal pathogens.

Many identification techniques have been developed and are currently used in clinical diagnosis, public health, animal welfare, plant protection, quarantine implications, and many other industrial fields (Figure 1). These conventional diagnostic tools vary in their success depending on the type of sample. However, despite advancements in molecular identification techniques of pathogens, these traditional methods still are an important component of routine diagnosis.

### Morphology

Morphology-based methods rely on the identification of phenotypic features of the pathogens at the macroscopic or microscopic level. Physical characteristics can be observed within patient samples through tissue staining. However, growth on media is often required for diagnosis (Guarner and Brandt, 2011; Austin, 2017). Fungal pathogens may be identified based upon simple characteristics, such as the color, size, and smell of the isolates. Often, these do not allow for complete diagnosis, as these features may be shared among many species. More in-depth observations are required, including the analysis of the complexity of morphology, the presence of sexual reproductive structures, the effect on surrounding media, and the effect of different staining techniques (Kurtzman et al., 2011; Schelenz et al., 2015).

### Biochemical Methods

Commercial diagnostic tests based on characteristic biochemical profiles produced by fungi are available for diagnostic use. These tests provide a positive or negative result for metabolic, enzymatic, or fermentation products produced by specific fungal species and so are suitable for confirmatory testing after a suspected pathogen has been found through more broad range methods (Chen et al., 2014; Lackner et al., 2014; Schelenz et al., 2015; Wagner et al., 2017).

### Serology

Serology-based tests identify the presence of antigens and antibodies within patient serum indicating current and previous infections (Im et al., 2019). Serological tests for fungal infections include testing for the presence of Galactomannan antigen for cases of invasive Aspergillosis (Pfeiffer et al., 2006; de Heer et al., 2019) and (1–3)-β-d-glucan (BDG) antigen testing for diagnosis of Candidemia and *Pneumocystis jirovecii* Pneumonia (Karageorgopoulos et al., 2011; White et al., 2017).

### Targeted PCR Amplification

Targeted amplification of DNA regions using species-specific primers by PCR has been widely employed for the identification of fungal pathogens (Kidd et al., 2019). A wide array of primers targeting the ITS1/2 region of the rDNA gene cluster has been designed for specific fungal species, including the prominent pathogenic fungal species *Candida*, *Aspergillus*, and *Fusarium* (Carvalho et al., 2007; Springer and Loffler, 2017). Additional regions have also been targeted by species-specific primers, e.g., the D1/D2 region of the large ribosomal subunit (LSU) of the rDNA gene cluster is used for the identification of *Candida* species (Mannarelli and Kurtzman, 1998) and for *P. jirovecii* (Tia et al., 2012). The latest targeted amplification-based identification method is the T2Candida Panel (T2 Biosystems), which is a new diagnostic tool for rapid, sensitive, and specific detection of invasive candidiasis from whole blood without culturing or nucleic acid extraction (Neely et al., 2013). It relies on PCR amplification and T2 Magnetic Resonance which enables nanoparticle-mediated rapid detection of candidemia with a sensitivity of one colony forming unit (CFU)/ml of whole blood (Mylonakis et al., 2015; Clancy et al., 2018). The use of species-specific PCR is limited in fungal diagnostics, as it is reliant on prior suspicion of the fungal pathogen to select the correct PCR target, primers, and amplification conditions (Wickes and Wiederhold, 2018).

### Matrix-Assisted Laser Desorption/Ionization Time of Flight Mass Spectrometry

Matrix-assisted laser desorption/ionization time of flight mass spectrometry (MALDI-TOF MS) is a high-throughput, reliable technology used to identify and analyze large biomolecules (Fenselau and Demirev, 2001; Clark et al., 2013). Starting material for MALDI-TOF can be found within sterile patient samples with high microbial load; however, this is not commonly possible and so prior pathogen growth is required. The processed material is then suspended in a crystalline matrix, vaporized, and ionized by a laser. Charged particles are then separated in a high voltage field, and the time of flight of the particles is measured and recorded as mass spectrum. Organism identification is obtained *via* the comparison of the obtained mass spectra with a reference database, which is currently the limiting factor (Angeletti, 2017).

Traditional identification techniques largely rely on prior growth of pathogens before testing may commence. This puts a strain on diagnosis as some fungal species may require up to 4 weeks before discriminatory features can be observed (Irinyi et al., 2016). Closely related species and species complexes, which may have different responses to antifungals, are unable to be reliably identified as characteristic phenotypic traits are not displayed and can only be genetically distinguished, such as in the genera *Fusarium*, *Aspergillus*, and *Scedosporium* (Goldstein et al., 2015; De Hoog et al., 2019). Traditional identification methods have been shown to be more insensitive than more advanced techniques, as results are highly subjective and error prone, leading to potential misidentifications (Table 1; Irinyi et al., 2016; Gustafson et al., 2019). Additionally, a number of fungal species, such as *P. jirovecii*, cannot be cultured under laboratory conditions and so culture-based methods are inappropriate for the identification (White et al., 2017). MALDI-TOF most closely meets the criteria for an ideal diagnostic test; however, it remains reliant on culturing and still lacks an exhaustive curated database. Although MALDI-TOF has been demonstrated to be more cost-efficient than traditional culturing techniques, the initial instrument cost limits widespread use in smaller diagnostic laboratories (Patel et al., 2017).

**Table 1:** Features of diagnostic tests available for fungal infections.

	Diagnostic test	Available since	Culture dependency	Need for single culture	Test turnaround time without culture time	Accuracy	Curated database needed	Expertise	Cost	Portability	Resolution power	Potential for virulence and drug resistance detection	Generated clinical information
Non-sequence-based	Morphology	1676	Yes	Yes	Long	Low	No	High	Low	No	Medium	No	Low
	Biochemical	1975	Yes	Yes	Long	Low	Yes	Low	Medium	No	Medium	No	Low
	Serological	2003	Yes	No	Short	Low	No	Low	Low	No	None	No	Low
	Targeted PCR amplification (T2Candida panel)	2013	No	No	Short	High	No	Low	Low	No	High	No	Low
	MALDI-TOF	2008	Yes	Yes	Short	High	Yes	Low	Low	No	High	No	Low
Sequence-based	PCR based assays	1977	Yes	No	Medium	High	No	Medium	Low	No	High	No	Medium	
	Sanger sequencing/DNA barcoding	1977	Yes	Yes	Medium	High	Yes	Medium	Medium	No	High	No	Medium	
	Whole genome sequencing	2005	Yes	Yes	Long	High	Yes	High	High	No	High	Yes	Very high	
	Long-read metagenomics/Metabarcoding	2015	No	No	Short	High	Yes	Medium	Low	Yes	High	Yes	Very high

*Overall, non-sequencing-based identification techniques are inadequate as the gold standard diagnostic technique for fungal infections.*

## Sequencing-Based Identification Techniques

The advancement in the understanding of the genetic information of microorganisms has been fundamental for the development of molecular diagnosis techniques of pathogenic fungi. These techniques include PCR – restriction fragment length polymorphism analysis (Dendis et al., 2003), random amplified polymorphic DNA analysis (Brandt et al., 1998; Mobasherizadeh et al., 2016), hybridization with genus/species specific DNA or RNA probes (Sandhu et al., 1995; Lindsley et al., 2001), PCR-fingerprinting (Lieckfeldt et al., 1993), species-specific PCR assays (Martin et al., 2000; Kulik et al., 2004), real-time PCR (Klingspor and Jalal, 2006), and increasingly DNA sequencing (Pryce et al., 2003; Romanelli et al., 2010). DNA sequencing introduced by Sanger et al. (1977) dominated the sequencing technology for 20 years, being recognized as first-generation sequencing. When applied to the identification of microorganisms, it relies on the analysis of pathogen-specific genome regions for comparison to reference sequence databases.

DNA barcoding is one of the most promising and efficient sequencing-based methods enabling rapid identification of species and recognition of cryptic species. The concept was first proposed by Hebert et al. (2003). DNA barcodes are standardized, easily amplified, universal, short DNA sequences (500–800 bp), which show a high divergence at the species level. These allow for the rapid identification of an organism though comparison of the generated genetic barcode to a reference collection of DNA barcodes from well-identified species. Two barcoding regions have been established for use in the identification of pathogenic fungi. The primary fungal DNA barcoding region is the internal transcribed spacer region (ITS1/2; Schoch et al., 2012) and the secondary fungal DNA barcoding region is the *translational elongation factor 1α* (Stielow et al., 2015). DNA barcoding using Sanger sequencing has major inherent limitations, including: (1) the requirement of a single fungal organism in the sample, (2) high target amplicon yield to avoid biases and errors (Polz and Cavanaugh, 1998), and (3) intra-individual variability (heteroplasmy). Especially, the high intra-genomic diversity of the ITS1/2 region in fungi cannot be simultaneously detected from one sample using Sanger sequencing alone, as samples with multiple amplicons will appear as mixed peaks in the sequencing chromatograms (O'Donnell and Cigelnik, 1997). The high intra-genomic diversity was only able to be detected after cloning (Jumpponen, 2003; Simon and Weiss, 2008) and the application of Next-Generation Sequencing (NGS) technologies (Ganley and Kobayashi, 2007; Colabella et al., 2021).

Sequencing technologies have recently rapidly advanced. The early NGS technologies were classified as second-generation sequencing technologies and achieved increased sequencing throughput after the amplification of thousands of DNA templates and the simultaneous sequencing of the resulting DNA fragments, saving time, and decreasing costs (Heather and Chain, 2016; Ravi et al., 2018). The most prominent short-read sequencing technology is those produced by Illumina. While Sanger sequencing suffered from low throughput, it did produce long-reads (up to 1,000 bp) with high accuracy. Short-read NGS technologies focused on achieving high throughput while sacrificing read length (100–600 bp; van Dijk et al., 2014; Illumina, 2021). The high throughput of NGS allowed for in-depth sequencing, recovering more data and thus more species from complex samples (Shokralla et al., 2014).

*The introduction of sequence-based identification methods improved the speed and accuracy of fungal diagnostics and the rapid advancements of sequencing technologies enables the identification of fungi in complex samples.*

## Metabarcoding and Metagenomics

Metabarcoding/metagenomic sequencing is a method in which nucleic acid (DNA or RNA) of all organisms in a sample is extracted and sequenced using NGS techniques. The generated sequences are then used to identify organisms present in the sample. It was first mentioned in 1998 (Handelsman et al., 1998), when it was applied for the culture independent analysis of a complex and diverse (“meta”) community of microorganisms.

There are two main approaches which can be used to characterize the microbiome of a specific sample: (i) **targeted amplicon sequencing (metabarcoding)** and (ii) **shotgun metagenomics**. While the amplicon sequencing is based on the amplification of well-characterized genetic regions, shotgun metagenomics attempts to sequence the entire genetic content in a sample. The detailed differences between the two approaches have been previously discussed elsewhere (Forbes et al., 2017).

**Targeted amplicon sequencing** (metabarcoding) enables the direct identification of multiple species simultaneously from any environmental or clinical sample without culturing. It has been extensively used in microbiome studies and its biggest advantage over shotgun sequencing is the low amount of microbial DNA needed for identification and targeted enrichment of the sequence of interest. It is based on the amplification of specific taxonomic genetic regions (barcodes), such as the 16S ribosomal RNA (rRNA) gene cluster for bacteria (Riesenfeld et al., 2004; Janda and Abbott, 2007) or the internal transcribed spacer region (ITS1/2; Nilsson et al., 2009; Schoch et al., 2012) for fungi, by universal degenerated primers in a PCR reaction. These barcodes are ubiquitous in varying copy numbers, can be easily amplified, and have two fundamental characteristics: high taxonomic coverage and high resolution (Schoch et al., 2012). This approach combines the concept of DNA barcoding (Hebert et al., 2003) and the application of high-throughput sequencing technology (metabarcoding). The generated barcodes are then computationally clustered by sequence similarity into operational taxonomic units (OTUs) and queried against a reference database, such as UNITE^1^ (Koljalg et al., 2005), BOLD^2^ (Ratnasingham and Hebert, 2007), RefSeq^3^ (Schoch et al., 2014), and the ISHAM Barcoding Database^4^ (Irinyi et al., 2015; Meyer et al., 2019). The targeted approach only sequences the amplified barcodes and not the whole genome. Due to the smaller size of the generated data, the computational analyses are less complex and faster. However, amplicon-based sequencing has PCR biases due to its inability to amplify all microorganisms across multiple taxa and it does not provide any additional characterization beyond taxonomic information.

**Figure 2:**
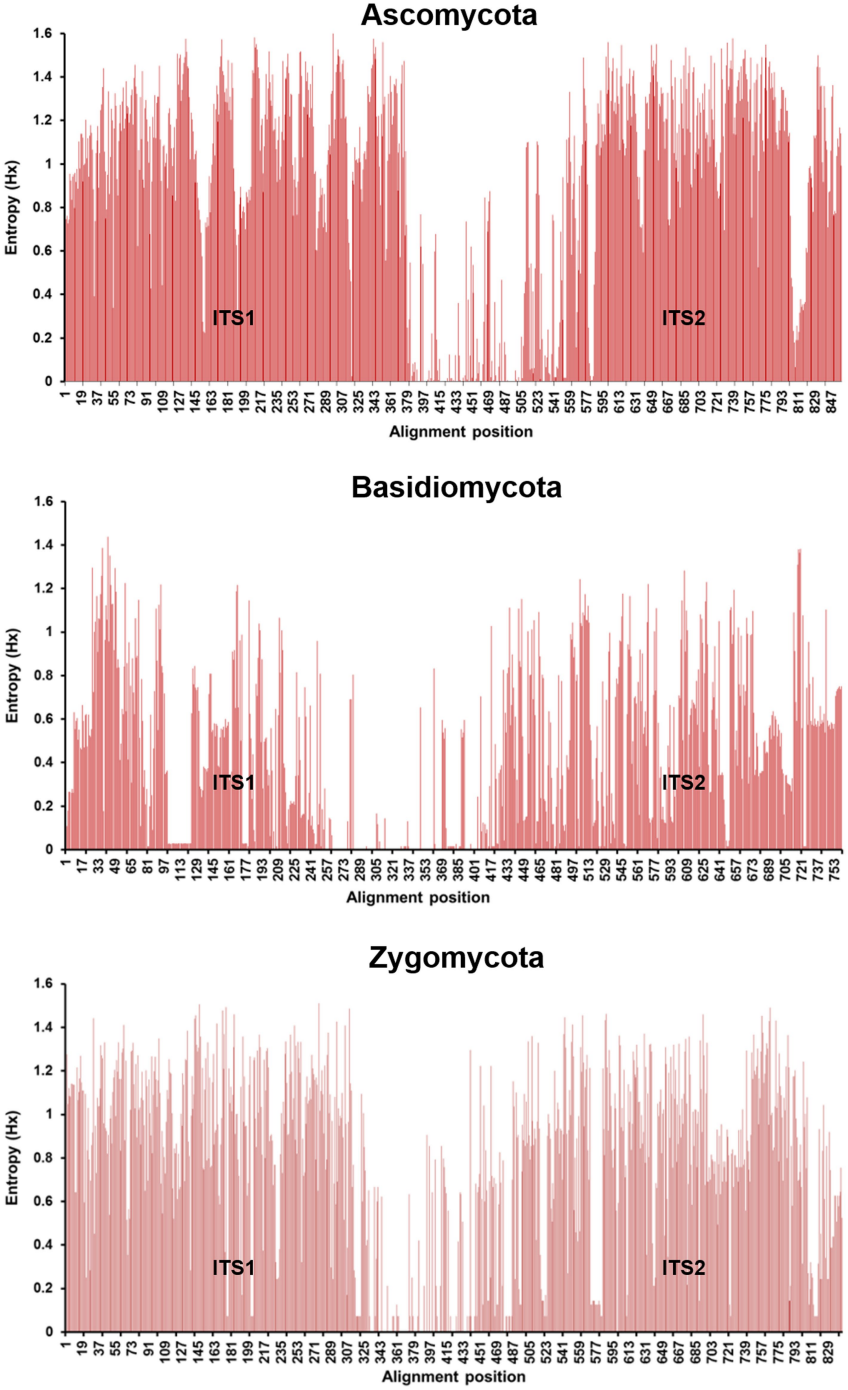
Nucleotide entropy plots showing the variations in the ITS1/2 region for Ascomycota, Basidiomycota, and Zygomycota. The higher the bar is the greater the variation at that position in the nucleotide sequence. The variation of ITS1/2 is higher than that of the ITS1 or ITS2 regions alone and so indicates higher discriminatory power for identification. Entropy values were calculated using BioEdit 7.0.

Short-read sequencing has been widely used in metabarcoding studies; however, it only targets short barcodes, such the ITS1 or ITS2 region for fungi, as second-generation sequencer read lengths (500–600 bp for paired and reads) are unable to span the full length of the established barcodes, the entire ITS1/2 region (Edouard et al., 2018; Hamad et al., 2018; Tsang et al., 2021). In addition, short-reads are unable to resolve repeated sequences that are longer than the reads generated by short-read NGS and as such these may lead to misassembles and gaps (Salzberg and Yorke, 2005; Treangen and Salzberg, 2011; Teng et al., 2017). Furthermore, even the short-reads lend themselves to identifying single-nucleotide variants and short indels accurately, longer structural variations are more challenging to resolve, as the variation is different within the ITS1 or ITS2 regions (Figure 2), leading to different discriminatory powers (van Dijk et al., 2018). As a result, in some species, the ITS1 or the ITS2 alone cannot resolve all species, which will only be possible using the combined ITS1/2 region (Irinyi et al., 2016). Longer sequencing reads would eliminate these issues and so drove the development of long-read NGS, see below.

In contrast, **shotgun metagenomic** sequencing allows for a higher resolution, as it sequences most parts of the genomes of every organism present in the sample. This feature enables the technology not only to identify the organism, but also to characterize their extended profiles, such as antimicrobial resistance, genetic subtypes, and virulence. It involves extraction of nucleic acid (DNA or RNA) from primary specimens (e.g., soil samples, water samples, blood, feces, bronchoalveolar lavage (BAL), and sputum, tissue), fragmentation of the obtained DNA or RNA extracts, library preparation, in-depth sequencing, and subsequent data analysis. Shotgun metagenomics is significantly more expensive (depending on the sequencing depth) and computationally more extensive than amplicon sequencing. Another challenge is the overwhelming amount of host or background DNA compared to the microbial DNA. Many methods have been developed for enriching microbial DNA to combat this for both environmental and clinical samples, see below. Additionally, in cases of non-sterile patient samples or co-infections, genomic material from multiple microbial species may prove difficult to assign correctly to the particular species present (Bharti and Grimm, 2021).

The application of shotgun metagenomics to diagnostics eliminates the need for culturing as identification would be made directly from patient samples (e.g., tissue, blood, feces, BAL, and sputum). As previously discussed, culturing greatly limits the detection of causative pathogens, as not all pathogens can be grown in a laboratory setting and it also greatly delays the timing of accurate identification (Garrido-Cardenas and Manzano-Agugliaro, 2017). In addition, metagenomics eliminates any sequencing bias that may occur as a result of PCR amplification (Wang et al., 2015). Comparing to amplicon sequencing, shotgun metagenomics also allows for better estimation of the relative abundance of microbes in samples based on the number of reads generated (Laudadio et al., 2018). Most importantly, shotgun metagenomics data contain additional genomic information from the patient samples, potentially informing not only on pathogen identities, but also underlying host factors. Previously established antimicrobial markers may also be identified from metagenomic data and as such can be used to guide targeted treatment, leading to the reduction of inappropriate use of antimicrobials and a subsequent reduction in the development of antimicrobial resistance. Additionally, further genomic information can be generated, to be used for epidemiological studies and outbreak tracing *via* the utilization of multi-locus sequence typing data generated as part of the metagenomics dataset (Couto et al., 2018).

*The quantity and quality of data generated from metagenomic studies is reliant upon the NGS technology used and as such the advantages and disadvantages must be considered. The characteristics of the main technologies used for the identification of fungal pathogens are summarized in* *Table 1*.

## Long-Read Sequencing Technologies

Third-generation sequencing technologies are characterized by the generation of ultra-long-reads and offer a number of advantages over short-read sequencing. Long-read sequencing routinely generates reads over 10 kb (Pollard et al., 2018; Mantere et al., 2019). Currently, commercial long-read sequencing is supported by two companies, Pacific Biosciences and Oxford Nanopore Technologies.

### Pacific Biosciences

Pacific Biosciences (PacBio) released the first commercial long-read sequencer in 2011 and continue to maintain and release improved sequencing instruments (Ardui et al., 2018; Tedersoo et al., 2021). The basis of Pacific Biosciences sequencers is known as single molecule real-time sequencing (SMRT), which takes place in single use SMRT Cells. These contain multiple Zero-mode waveguiders (ZMWs) that hold an immobilized polymerase. Double-stranded DNA (dsDNA) is prepared for sequencing by forming SMRT bells by adding hairpin adaptors to both ends of the dsDNA template (Eid et al., 2009). Each SMRT bell is diffused into one ZMW and nucleotides with identifying fluorescent labels are added to the ZMW alongside reagents required for PCR. From there, the immobilized polymerase binds to the hairpin adaptor of the SMRT bells and begins replication. As a nucleotide is added to the complementary strand, fluorescence at a characteristic wavelength is emitted and the sequence of fluorescence pulses is recorded into a movie. The wavelengths emitted are then converted into a nucleotide sequence called a continuous long-read. After the polymerase completes replication of one DNA strand, it continues to sequence the opposite adapter and second strand. As a result, it is possible to generate multiple passes of the same template depending on the lifetime of the polymerase. Upon sequencing completion and conversion to continuous long-reads, subreads are formed by identifying and cleaving the adaptor region sequences. Multiple computationally merged subreads of the same template DNA strand are called circular consensus sequences or HiFi reads, and the more subreads, the higher the accuracy, currently reaching up to Q30–50 for consensus HiFi (Rhoads and Au, 2015; Tedersoo et al., 2021).

PacBio currently have three sequencers on the market, the Sequel System, the Sequel II system, and the Sequel IIe system, released in October 2020 (Pacific Biosciences, 2020). The newer iterations Sequel II and Sequel IIe boast a new SMRT Cell, dubbed the SMRT Cell 8 M, containing 8 million ZMWs capable of running for 30 h and generating up to 4,000,000 reads with over 99% (Q20+) accuracy (Wenger et al., 2019; Pacific Biosciences, 2021a). The Sequel IIe improves upon the Sequel II by upgrading the software and data analysis function through data processing capability on the instrument and cloud facilitation leading to potentially 70% reduction in secondary analysis time and 90% reduction in file transfer and data storage (Pacific Biosciences, 2020).

SMRT sequencers initially reported low read accuracy of 85–87%, when at the same time, short-read sequencers from Illumina had read accuracy >99% (Chin et al., 2013; Ardui et al., 2018). Errors in sequencing through insertions and deletions were more common in SMRT sequencing. However, improvements in sequencing chemistry and polishing methods increased the read accuracy of SMRT sequencing to 99.8% (Ardui et al., 2018; Tedersoo et al., 2018; Wenger et al., 2019). This allowed its use in direct sequencing studies rather than only be used to scaffold short-read Illumina reads (Parker et al., 2017; Teng et al., 2017; De Maio et al., 2019). The increased throughput of SMRT sequencers has made adequate coverage with multiplexed samples achievable (Armanhi et al., 2016; Pacific Biosciences, 2021b). The current SMRT Cell 8 M supports the simultaneous sequencing of 348 samples when using the largest barcoding kit PacBio currently offers, and the sequencing depth can achieve reference quality for *de novo* assemblies for genomes up to 2GB of a single sample and full length 16S region sequences for 192 samples for strain level identification (Pacific Biosciences, 2021b).

Disadvantages of PacBio instruments are, that they require a high initial investment cost, limiting widespread use. The throughput of Illumina sequencers remains higher than PacBio, as PacBio sequencers are limited by the number of ZMWs available in the SMRT Cells. Not all ZMWs are guaranteed to successfully sequence, as there maybe more than one DNA fragment being present in a ZMW, and there maybe issues with the polymerase binding. The nature of SMRT bells results in a trade-off between read length and read quality as longer DNA inserts are sequenced less often and hence the generated consensus sequence is of lower quality (Tedersoo et al., 2018). Further, the library preparation for the Sequel II Instrument is estimated to be 1 day, far longer than needed for the Oxford Nanopore Technology library preparation kits (see below), which may not be suitable for time sensitive diagnostic applications.

*Overall, Pacific Biosciences has successfully created sequencing instruments capable of generating high accuracy ultra-long-reads.*

### Oxford Nanopore Technologies

The idea of nanopore-based technologies originated from the Coulter counter and ion channels (Cornell et al., 1997). The single molecule sequencing using biological nanopores was first proposed in 1995 and published in 1998 (Church et al., 1998). Kasianowicz et al. (1996) than described the detection of ssDNA passing through an α-hemolysin nanopore (2.6 nm in diameter; Kasianowicz et al., 1996). Oxford Nanopore Technologies (ONT) officially launched the concept of MinION™ in 2012 as a new generation nucleic acid sequencing technology based on nanopores, free from florescence labels and amplification requirements. It became commercially available in 2015 (Eisenstein, 2012; Deamer et al., 2016). The MinION™ is a palm sized portable single molecule sequencing device capable of generating high-throughput, ultra-long sequence reads in real-time at relatively low cost.

The sequencing chemistry of ONT’s sequencers has undergone rapid development with the latest flow cell released at the time of writing being the R10.4. However, the basis of nanopore sequencing remains consistent, relying on biological nanopores embedded in solid state membranes within disposable flow cells (Leggett and Clark, 2017). Different sequencing library preparation methods are available depending on the intended use and data requirements. However, in all library preparation methods, a leader adapter and motor protein are ligated to the end of dsDNA fragments. This library is then loaded into the flow cells and the motor protein functions to unzip dsDNA and process the DNA strand throughout the pore at a specific speed. After one strand is processed, the pore is available to sequence the next available strand. Nucleotides are processed through the biological pores in groups of four called k-mers and as these travel through the pore, a change in an ionic current across the membrane occurs. These changes are then recorded as signal traces and during base calling, are converted to nucleotide sequences (Jain et al., 2016).

ONT’s MinION flow cell contains 4x512 nanopore channels, each containing one nanopore, with 512 channels being accessed at a given time. These are run on both the MinION, which supports one flow cell at a time, and the GridION, which supports five flow cells. The PromethION is ONT’s production-scale sequencer and provides 3,000 nanopore channels for each flow cell (Oxford Nanopore Technologies, 2021). All instruments can be run until the flow cells no longer have any pores available for sequencing and can be stopped when the intended sequencing depth is achieved. Additionally, the Flongle is a MinION adapter that enables the use of Flongle flow cells that contain 126 nanopore channels at a reduced cost (Oxford Nanopore Technologies, 2019).

The primary appeal of ONT sequencers over other current sequencing technologies is that read length is mostly dependent on sample DNA length, besides high DNA quantity and purity. It has been previously demonstrated to generate read lengths of over 2 million base pairs (Payne et al., 2019). ONT sequencers also offer reduced initial investment costs for sequencers when considering the MinION and the Flongle adapter although library preparation costs remain high (Tedersoo et al., 2021). Per sample sequencing costs depend on multiplexing, sequencing coverage, and re-use of flow cells after washing with a DNase. This makes sequencing available to a wider range of small routine laboratories when other sequencers may only be available for large laboratories or sequencing center. The availability of higher throughput instruments allows nanopore sequencing to be scaled to many different purposes. Multiplexing kits of up to 96 samples are available to further scale projects down if less in-depth sequencing is needed or a lower cost-per sample is necessary. ONT sequencers are highly suited for use in unconventional settings, as field library preparation kits are available that reduce library preparation time to 10 min with limited laboratory equipment. The sequencers are portable as the MinION and Flongle adapters are palm sized, allowing sequencing to be done outside the laboratory, such as at disease outbreak hotspots, war zones or for real-time environmental monitoring. This has previously been demonstrated through its use in space and resource poor settings (Quick et al., 2016; Castro-Wallace et al., 2017). The major drawback of nanopore sequencing has been the low read accuracy being reported with 90% with the R9.4 flow cell and earlier models (Lu et al., 2016). The introduction of new flow cells has led to strong improvements, with average accuracies of 87–98% being commonly reported (Loit et al., 2019; Logsdon et al., 2020). The highest accuracy ever reported after bioinformatics polishing, which might be computation-intensive and may take weeks, stands at >99% (Ashikawa et al., 2018; Morrison et al., 2020). In addition, new bioinformatic tools to improve the accuracy of nanopore reads are now readily available (Loman et al., 2015; Koren et al., 2017; Salmela et al., 2017; Xiao et al., 2017; Hu et al., 2021). As sequencing quality and length depends on the quality of the applied DNA/RNA samples a major drawback is that impurities within the loaded library may block pores and render them unable to sequence.

*Overall, nanopore sequencing is by now a broadly accessible long-read sequencing platform with read length limited only by the DNA input making it a fundamentally different approach for sequencing.*

## How Can Long-Read Sequencing Be Applied To Clinical Diagnosis

As long-read sequencing platforms have the potential to accurately identify pathogenic species and simultaneously provide additional genomic information impacting patient outcome, we are focusing the remaining part of the review on the ONT and PacBio platforms for their use in clinical diagnostics.

The read length of SMRT sequencers has advanced to where it is comparable to those generated by ONT although read length is determined by input DNA for both sequencers. When using these directly on patient samples without prior amplification, as in metagenomics, long-reads provide genetic information spanning the whole informative regions of pathogen genomes. For example, DNA barcoding regions, which are between 500 and 800 bp, cannot be covered by short-read sequencing read lengths but are now being able to be fully sequenced by the long-read sequencing instruments. It would also be possible to sequence antimicrobial genes present in the genomes of resistant microbes, informing treatment strategies to improve patient outcome. Long-reads may also allow accurate sequencing of loci important in surveillance or strain typing. In samples where pathogen material is low in abundance, a prior amplification step of important genetic regions would ensure the sequencing of these important target regions although potentially missing other information.

The application of metagenomics to clinical diagnosis requires reliable and highly accurate sequencing and both ONT and PacBio have advertised over 99% per read accuracy for their latest sequencing chemistries. For PacBio, this has been reflected in many recent studies, e.g., Wenger et al., 2019. The accuracy of ONT depends on the type of sequencing chemistry and base-calling algorithms used. The accuracy of ONT technologies has been significantly improving and high accuracy levels are targeted to be achieved in the near future. Although nanopore sequencing may not yet be suitable for applications, such as single-nucleotide variant identification or genotyping, which requires high coverage to exclude false positives, however, the accuracy required for pathogen identification can already be achieved (Greig et al., 2019; Feng et al., 2021).

Sequencing requirements of diagnostic laboratories cannot be guaranteed to be constant and so this may result in different throughput requirements. As such, investments into high-throughput sequencing instruments, such as the PacBio instruments, are not suitable for routine diagnostic laboratories. However, an alternative may be the implementation of core diagnostic centers that service multiple smaller laboratories and the multiplexing of samples, which would provide an effective way to reduce the cost-per sample. However, this would result in waiting for an adequate number of samples to be pooled for sequencing, which is contradicting the fact that fast diagnosis being the most important facture for the best patient outcome. As such, the smaller scale sequencing offered by ONT with the Flongle flow cells allows for sequencing of individual samples when needed, which is more suitable for general routine laboratories. Additionally, the initial capital investment in sequencing instruments, such as the ones produced by PacBio, may not be feasible for smaller diagnostic laboratories, which only could be overcome by pooling resources into larger diagnostic centers. The smaller scale ONT sequencers are generally affordable, making them more suitable in low throughput settings, such as it is the case in the diagnostic setting for mycoses.

**Figure 3:**
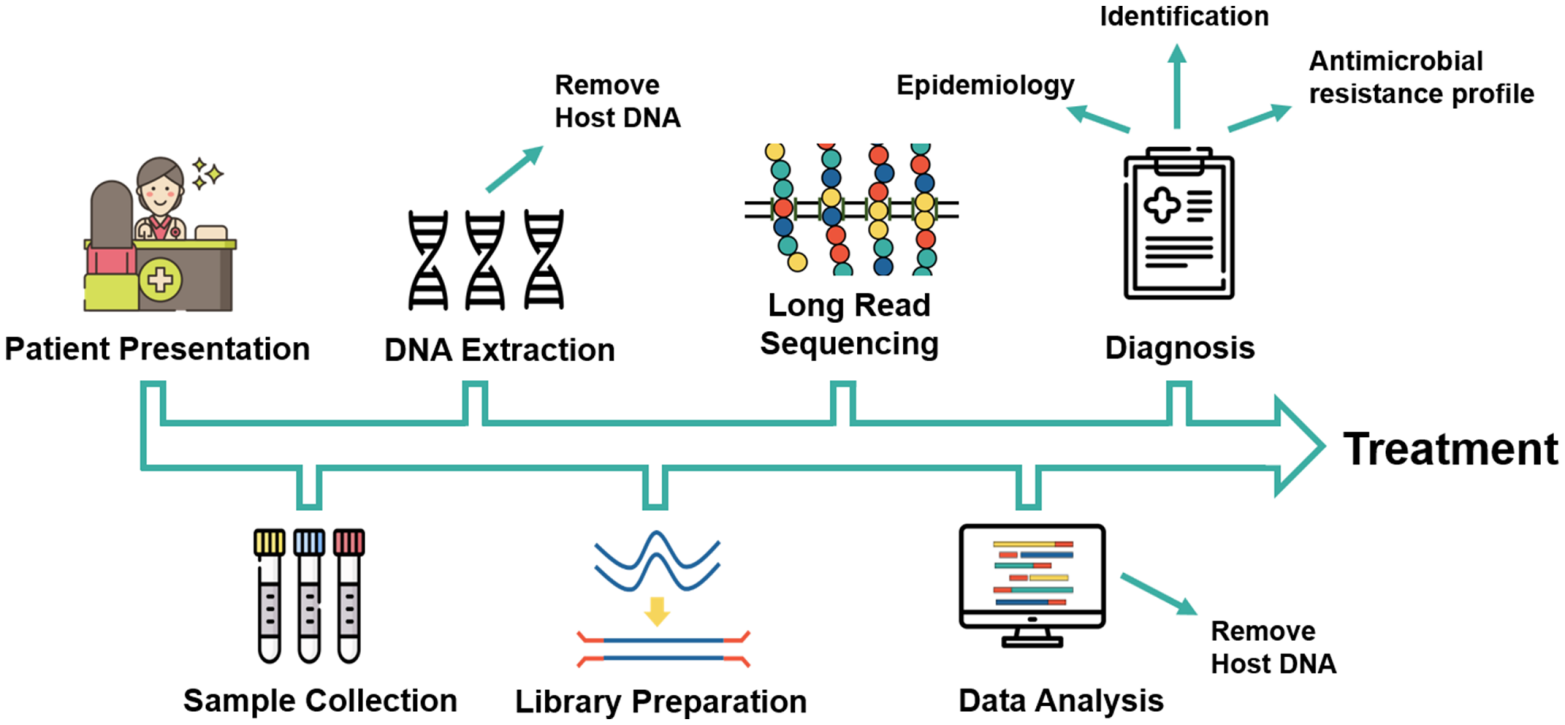
Proposed workflow for long-read-based sequencing in clinical diagnosis of fungal diseases. Icons made by https://www.flaticon.com/authors/freepik.

The proposed workflow of the use of long-read sequencers in clinical diagnosis illustrates the need for timely identification (Figure 3). The time from sample collection to identification must be as rapid as possible and so the selection of a sequencer and its sample preparation and sequencing time must be considered. PacBio sequencing reports library preparation time of 1 day, while the slowest ONT library preparation kit, the direct cDNA Sequencing Kit, has an estimated preparation time of 270 min. The shortest library preparation option for ONT sequencing is the Field Sequencing Kit (SQK-LRK001) and the Rapid Sequencing Kit (SQK-RAD004), which have a preparation time of only 10 min although these kits produce a lower throughput (Kafetzopoulou et al., 2018). There are also further efforts to reduce turnaround time by reducing sample preparation and sequencing times (Loit et al., 2019). Further sample preparation may be added, which increases preparation times, such as a prior amplification step and sample multiplexing. Both PacBio (Sequel IIe) and ONT provide the option for real-time sequencing, thus eliminating the need to wait for the sequencing to be completed before base-calling can occur. This allows for a substantial reduction of the time from sample collection to pathogen identification to less than 24 h, enabling a faster introduction of the correct treatment, and improving patient outcome.

Before long-read sequencing can be applied to routine diagnostic testing, further work is required to determine the accuracy, specificity, and precision, of long-read sequencers in a clinical setting. The limit of detection must also be determined before it can be reliably implemented. In addition, adequate DNA isolation, library preparation, and loading according to standardized procedures performed by trained personnel are required to take full advantage of long-read technologies for routine diagnostics laboratories.

*Long-read sequencers have many features that make them an attractive alternative for the use in clinical diagnosis of infectious disease agents.*

## Long-Read Sequencing Applied To Infectious Disease Investigations

The application of long-read sequencers to bacterial and viral species identification within a clinical setting is more advanced than that for fungal infections. The general smaller size of bacterial and viral genomes enables to use whole-genome sequencing studies instead of or in parallel with metagenomics studies, with both employing shotgun sequencing methods without prior amplification. As such, whole-genome sequencing studies will be discussed below alongside metagenomics and metabarcoding studies using long-read sequencers.

### Bacterial Infections

The ability of PacBio to identify bacteria using the full length 16S region to the species level was demonstrated by the study by Earl et al. in which 100% of a mock community of 20 bacterial species was identified to the species level (Earl et al., 2018). This was then extended to apply sequencing of a mock community of 250 bacterial species, of which over 90% were accurately identified to the species level. Finally, it was successfully applied to characterize the microbiome of six sinonasal sites of 12 subjects (Earl et al., 2018). The use of PacBio in identification of bacterial species through sequencing of the full length 16S rRNA gene was further demonstrated through generation of microbiome profiles for vaginal samples and sputum samples from patients with cystic fibrosis (Hahn et al., 2016; Wagner et al., 2016). PacBio long-reads have also been used in the generation of whole-genome reference sequences of clinical samples indicating its potential use for future metagenomic studies to identify pathogenic bacteria (Kanamori et al., 2016; El-Rami et al., 2019).

Metagenomics and metabarcoding studies using nanopore sequencers for bacterial samples have been widely used in clinical and research settings. Rapid and accurate diagnosis of a *Capnocytophaga canimorsus* infection in a 62-year-old female patient using metagenomic nanopore sequencing has been reported (Bialasiewicz et al., 2019). This study demonstrated a positive identification was achieved by nanopore sequencing within 19 h, whereas conventional diagnostic techniques required 6.5 days to achieve the same. This short turnaround time from beginning of the sample preparation to pathogen identification is reflected in other studies, estimating a 6–8-h turnaround time (Schmidt et al., 2017; Charalampous et al., 2019; Gargis et al., 2019; Greig et al., 2019), and even using as little as 30 min of sequencing (Sakai et al., 2019; Taxt et al., 2020). Gu et al. (2021) also found a turnaround time of 6 h for nanopore sequencing compared to 24 h to Illumina sequencing. The study also demonstrated accurate identification of pathogenic bacterial species from a variety of clinical samples using metagenomic nanopore and Illumina sequencing. These samples also ranged from culture and PCR negative to PCR positive. In cases where NGS was unable to identify the causative pathogen, the cause was likely due to low pathogen titer or high levels of host background (Gu et al., 2021). An appeal of the use of metagenomics in clinical diagnostics is the potential for additional genomic information, such as antimicrobial resistance coding genes, to be obtained during sequencing. This has been achieved in studies for common bacterial pathogens, such as *Echerichia coli*, *Klebsiella pneumoniae*, *Mycobacterium tuberculosis,* and *Staphylococcus aureus* (Schmidt et al., 2017; Votintseva et al., 2017; Cheng et al., 2018; Charalampous et al., 2019; Gonzalez-Escalona et al., 2019; Taxt et al., 2020). Metagenomic sequencing of clinical bacterial samples has also revealed accurate epidemiological information that can be used for surveillance and outbreak tracing (Votintseva et al., 2017; Gorrie et al., 2018; Imai et al., 2020). ONT sequencers have also been used in metabarcoding studies involving the full length 16S rRNA gene to characterize complex mock communities and identify causative pathogens of clinical samples (Kai et al., 2019; Moon et al., 2019; Nakagawa et al., 2019; Winand et al., 2019).

*Many published studies applying long-read sequencing to the identification of pathogenic bacterial species in clinical samples strongly suggest its potential incorporation into routine bacterial diagnosis and encouraging its potential application in fungal diagnoses.*

### Viral Infections

PacBio sequencing has been used for whole-genome sequencing of a variety of human pathogenic viruses. A limitation of sequencing of viral genomes from clinical samples is the low viral load within cells. As such, reaching the required coverage to ensure an accurate consensus is a challenge. The small size of viral genomes lends themselves to overcome this issue through the amplification of the viral genome, which has been successfully used in whole-genome sequencing studies using PacBio, where the read lengths are able to span the whole amplicon length (Ocwieja et al., 2012; Dilernia et al., 2015; Bull et al., 2016; Nakano et al., 2017). PacBio sequencing has been demonstrated to be capable of the identification of viral pathogens from clinical samples but has not yet been used in routine viral diagnosis due to the high costs associated (Ho et al., 2017; Li et al., 2020). Identification of norovirus from patient samples has been performed to simulate diagnosis during an outbreak and demonstrated PacBio achieved sequencing of the entire viral pathogen, Norovirus GII, with high coverage of 99.11%; however, this was greatly limited by the long turnaround time (66 h; Li et al., 2020). PacBio reads have also been utilized in sequencing SARS-CoV-2 from patient samples to study the evolution and progression of disease and inform antiviral treatment (Ko et al., 2021). PacBio has additionally been used to achieve deep sequencing of genomic regions of viruses that are unable to be resolved using short-read sequencers (Bergfors et al., 2016; Huang et al., 2016; Ho et al., 2017; Lui et al., 2019; Takeda et al., 2019; Yamashita et al., 2020). Although these studies do not aim to identify the pathogens, the regions targeted for deep sequencing inform on resistance to antiviral treatments.

ONT has been widely used for the identification of viral pathogens in disease diagnostics and surveillance. Like the PacBio studies, nanopore sequencing of viral genomes often first involves either the amplification of the full genome or the generation of overlapping pooled amplicons that span the entirety of the genome (Quick et al., 2017; Deng et al., 2020). Nanopore sequencing has been demonstrated to be a useful tool for the identification of major pathogens during outbreaks, such as for the Ebola epidemic in West Africa from 2014 to 2016 and to generate additional genomic information on geographic, climate, and demographic factors (Hoenen et al., 2016; Quick et al., 2016; Dudas et al., 2017). Nanopore sequencing has also been used in the recent COVID-19 pandemic, e.g., for a prospective study of the complex transmission within healthcare settings through the generation of 747 SARS-CoV-2 genomes from PCR-positive diagnostic samples within 24 h of sample collection (Meredith et al., 2020). Epidemiological information of the spread of a variant of SARS-CoV-2 was generated using nanopore sequencing to inform on further action needed to control its spread (Washington et al., 2021). ONT sequencers have additionally been used for whole-genome sequencing during the Zika virus epidemic (Faria et al., 2017), Yellow fever virus epidemic (Faria et al., 2018), and the Lassa fever outbreak in Nigeria (Kafetzopoulou et al., 2019). Whole-genome sequencing from clinical samples has also been demonstrated for human metapneumovirus (Xu et al., 2020), Chikungunya and Dengue viruses (Kafetzopoulou et al., 2018), Influenza (Lewandowski et al., 2019), and Hepatitis C virus (Greninger et al., 2015).

*Long-read sequencing technologies have demonstrated their ability to be used to diagnose viral infections in the clinical setting and have a proven track record for analysis of samples during epidemics and a pandemic to produce valuable public health information.*

### Fungal Infections

The application of long-read sequencing to fungal identification in a clinical setting is currently limited.

Whole genomes of major fungal pathogens have been generated with the use of long-read sequencers often in combination with short-read sequencing, so called as hybrid assemblies (Cuomo et al., 2017; Luo et al., 2017; Vale-Silva et al., 2017; Panthee et al., 2018; Rhodes et al., 2018; Morand et al., 2019; Schultzhaus et al., 2019; Pchelin et al., 2020). The use of whole-genome sequencing in clinical practice is not yet feasible, as the cost and data processing requirements are currently too high for routine use.

In clinical applications, PacBio sequencers have been used to characterize the fungal composition of fecal samples from 14 healthy individuals (Motooka et al., 2017). The study identified multiple fungal species present within the healthy gut mycobiota and characterized two major mycobiota types. This study by Motooka et al. (2017) additionally found PacBio was able to accurately identify all 26 fungal species in a mock community through metabarcoding of the ITS1 region, where short-read sequencers could not. Additionally, PacBio most accurately estimated the abundance of species within the mock community. The predominant reason PacBio outperformed the short-read sequencers was the use of long-reads, ensuring complete and accurate coverage of the entire 300–800 bp long ITS1 region (Motooka et al., 2017). PacBio has also been used for metabarcoding analysis of environmental fungal samples using the shorter ITS2 minibarcode and the large ribosomal subunit (28S), which are often used by short-read barcode studies (Cline and Zak, 2015; Kyaschenko et al., 2017). Sequencing of the full length ITS1/2 DNA barcoding region by PacBio sequencers has been demonstrated on a wide variety of fungal species (James et al., 2016; Walder et al., 2017; Heeger et al., 2018; Tedersoo et al., 2018; Purahong et al., 2019; Tedersoo and Anslan, 2019). A comparison between PacBio and nanopore sequencers found PacBio to be more efficient in the metabarcoding of complex environmental fungal samples due to errors found in nanopore sequencing (Loit et al., 2019). Additionally, sample to identification time was reduced to 2.5 h through metagenomic nanopore sequencing of plant samples (Loit et al., 2019). These studies indicate the further potential for long-read PacBio sequencing to be used in metabarcoding to identify causative pathogens in clinical samples.

The use of ONT in metabarcoding and metagenomics is similarly limited; however, direct clinical use has been previously demonstrated (Irinyi et al., 2020; Gu et al., 2021). Three patient samples, positive for *P. jirovecii* infection, alongside three samples without infection underwent metagenomic sequencing using ONT’s MinION flow cells (Irinyi et al., 2020). The resulting reads were assigned using two different analysis tools, ONT’s own “What’s in my pot” and nucleotide BLAST, and the resulting assignments were compared. Sequencing of all three positive patient samples produced *P. jirovecii* reads while surprisingly, *Pneumocystis* reads were detected in negative samples when using the “What’s in my pot” analysis tool; however, this is attributed to issues applying the analysis tool to fungal identification. *Homo sapiens* sequences accounted for 70–95% of all reads besides one outlier (10%). Additionally, the sample sites were bronchiolar lavage and induced sputum samples which are non-sterile sites and so multiple other fungal species were identified alongside the known causative pathogen. Notably, *Paracoccidioides* spp. were identified within the sample. However, this indicates a clear false positive, as *Paracoccidioides* spp. are geographically restricted to the tropical areas of Latin America, which did not align with the patient’s travel history. This indicated that fungal diagnosis solely based off metagenomic sequencing using ONT has currently a number of limiting factors, including the low host:pathogen ratio, lack of comprehensive reference sequence databases, and the lack of appropriate bioinformatic tool, which need to be overcome before it can be applied in a routine diagnostic setting (Irinyi et al., 2020).

Gu et al. (2021) evaluated the diagnostic accuracy and performance of nanopore and Illumina sequencing on clinical body fluid samples (Gu et al., 2021). This study sequenced 87 patient samples by both sequencing technologies to compare the sensitivity and specificity. For fungal pathogen detection, the sensitivity and specificity of nanopore sequencing were found to be 90.9 and 100%, respectively, for nanopore sequencing, compared to 90.6 and 89.0% for Illumina sequencing. Additionally, five patient samples with negative culturing and PCR testing results were analyzed using NGS and three samples were found to be positive for fungal pathogens. For two samples, NGS did not detect any pathogens. However, the fungal pathogens *Cryptococcus neoformans* and *Sporothrix schenkii* were identified in these samples through further testing, most likely due to low pathogen titers or high host background DNA in the sample. This study demonstrated that mNGS can be applied to infectious disease diagnosis and would be a valuable tool in the routine diagnostic process (Gu et al., 2021). Even though this study achieved the identification of causative pathogens using ONT sequencing, it also highlighted again many issues currently associated with practical implementation into routine diagnostics, including the importance of the quality of the input DNA, the proportion of pathogen DNA to host DNA, and the lack of comprehensive reference databases (Gu et al., 2021). Furthermore,

Metabarcoding for the identification of fungal pathogens from clinical samples using nanopore sequencing has also been performed. The ITS1/2 region was amplified in a patient sample previously confirmed having a *Candia albicans* infection and the resulting amplicons were sequenced on the ONT MinION (Ashikawa et al., 2018). This resulted in 97.3% of the first 4,000 reads and 99.1% of reads generated after 48 h being assigned to *C. albicans*, confirming the previous diagnosis. Another study used metabarcoding by nanopore sequencing to identify 16 members of an artificial fungal community using the ITS1/2 region (Mafune et al., 2020). The ITS1/2 region was also used to identify 87% of the fungal species present in 43 respiratory samples and identified others not detected through routine testing (Chan et al., 2020). Metabarcoding of 1,312 clinical respiratory samples with the bacterial 16S gene and fungal ITS1/2 region identified pathogens in 51.5% of samples with a turnaround time of <24 h. Nanopore sequence alone identified 31% of samples, while 0.5% were identified with culture-based techniques only (Wang et al., 2020). Long amplicon nanopore sequencing of the full ITS region and the whole fungal operon successfully identified all members of a mock community and microbial cultures (D’Andreano et al., 2021). The full ITS region was also used to characterize the fungal microbiota of healthy and potentially infected clinical canine samples (D’Andreano et al., 2021). The fungal intergenic spacer region has also been demonstrated to identify members from the *Cryptococcus gattii* and *Cryptococcus neoformans* species complexes by multiplexing 24 strains on a single MinION flow cell. Sequencing errors in regions with homopolymers were observed. However, with the R10.3 flow cell, this error rate was reduced by 57% and the sequence identity increased to 99.83% (Morrison et al., 2020).

*These preliminary studies indicate the potential for the application of ONT and PacBio sequencers to the clinical diagnosis of fungal infections if the above-mentioned limitations are overcome to develop a workflow for clinical diagnosis.*

## Current Issues with Long-Read Sequencing

Long-read sequencers have been demonstrated to effectively identify bacteria, viruses, and fungi, from clinical samples using both metagenomics and metabarcoding approaches. Although these studies have illustrated the potential for long-read sequencing in clinical diagnosis, a range of current challenges facing their implementation has also been highlighted.

### DNA Extraction

Sequencing success depends on sample DNA quantity and quality. Pathogens are often present at low concentrations during infection and so high DNA concentrations are optimal to ensure adequate sequencing coverage of the causative pathogen for identification. Metagenomics and metabarcoding sequencing are aimed to represent everything within a sample and so ideally genomic material will be directly representative of what is present. As such, DNA extraction methods must be able to effectively lyse all cell types and preserve all genomic material.

For fungal pathogens, this presents a challenge, as fungal species have thick cell walls, containing polysaccharides and high content of lipids, that are difficult to lyse, which impedes the release of nucleic acids. As such fungal DNA extraction methods often involve a manual disruption step, such as bead bashing or crushing with liquid nitrogen to break open the fungal cell walls. This often leads to DNA shearing, greatly reducing the lengths of DNA fragments, defeating the purpose of long-read sequencing. A number of methods describing the extraction of DNA from fungal material for the use of nanopore and PacBio sequencing have reported to produce DNA fragments with an average fragment size above 35 k bp, although this has not yet been applied to clinical samples (Feehan et al., 2017; Schwessinger and Rathjen, 2017; Inglis et al., 2018). The development of better extraction methods is important to improve the application of long-read sequencers to accurate clinical diagnosis.

### Ratio of Host to Pathogen DNA

A major problem in metagenomics analysis is the high amount of host (human) DNA in the sample, which may shadow the detection of pathogen DNA. One possible solution would be to increase the proportion of pathogen DNA in comparison with host DNA within the sample. This may be achieved through the depletion of human DNA in the clinical sample, which still is suboptimal even though there are several commercial kits and laboratory protocols for this purpose available (Marotz et al., 2018; Charalampous et al., 2019; Helmersen and Aamot, 2020; Heravi et al., 2020).

An alternative to this would be to increase the abundance of pathogen DNA, which can be achieved through amplification of important genomic regions, such as in metabarcoding studies, or amplification of overlapping DNA regions as done in viral whole-genome sequencing (Quick et al., 2017; Deng et al., 2020). An improved host to pathogen DNA ratio would greatly improve identification using long-read sequencing as it would enable higher coverage of the causative pathogen, leading to more accurate identification and the possibility of further genomic information. An increase in pathogen reads would also make long-read sequencing more cost-effective since more relevant data would be generated, possibly allowing for multiplexing of samples.

Another way to enrich pathogen DNA and deplete host DNA is the application of adaptive sequencing with nanopore sequencers. DNA molecules are selected for by identifying unwanted sequences and reversing the voltage across the membrane, thereby stopping sequencing. Bioinformatic tools have been developed to implement adaptive sequencing for the selection of specific chromosomes and the depletion of bacterial genomes to enrich the remaining species in a metagenomic population (Kovaka et al., 2020; Payne et al., 2020). For diagnosis of mycoses, fungal DNA would ideally be selected for while human DNA is rejected; however, this is yet to be explored. The improvement of host to pathogen DNA ratio would greatly increase the amount of relevant data for clinical diagnosis and further work is required to implement these methods to routine diagnostics.

### Lack of Reference Genomes

**Figure 4:**
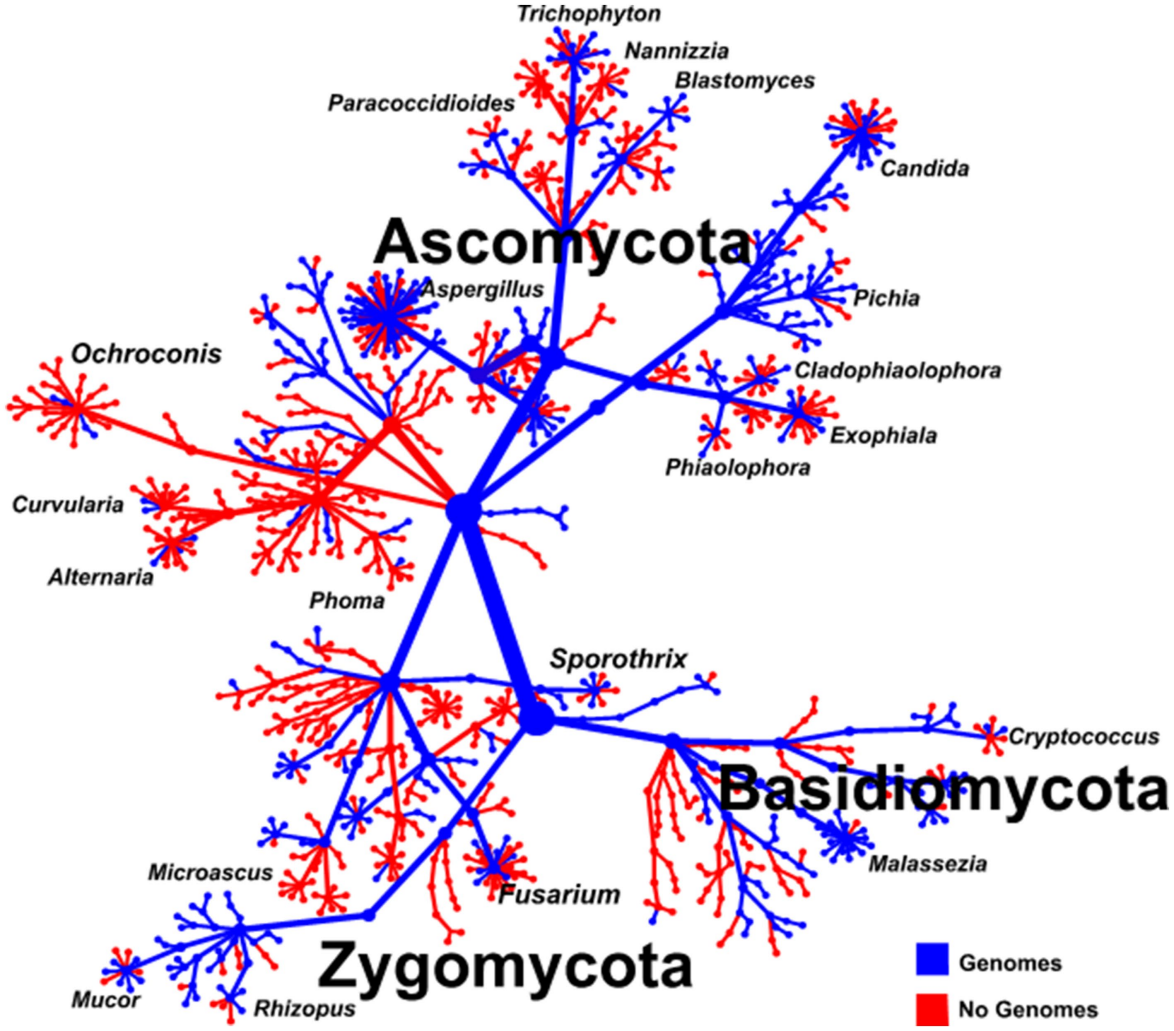
Tree of the fungal Kingdom showing the major fungal classes and their human pathogenic species indicating species with (blue) and without (red) genomes.

The major appeal of long-read sequencing in a shotgun approach is the vast genomic information that can be gained from the data generated. In metagenomic studies, genomic information from throughout the genome is generated; however, there is a lack of reference genomes for most of the pathogenic fungal species (Figure 4) and as such a proportion of data generated cannot be mapped to an appropriate reference genome either in FungiDB^5^ (Basenko et al., 2018) and GenBank^6^ (Sayers et al., 2020).

The basis for accurate genomic-based identification is the availability of high-quality, curated reference whole-genome databases. It has been demonstrated that inaccurate reference sequences have caused miss-mapping of the generated sequence reads leading to clear misidentification that potentially causes confusion surrounding diagnosis in clinical practice (Bialasiewicz et al., 2019; Irinyi et al., 2020). In addition, it is causing an unproductive strain on bioinformatic tools used and lengthening the time for identification. Diagnostic whole-genome database entries must have accurate sequences, metadata, and phylogenetic coverage to prevent false positive and negative identifications.

**Figure 5:**
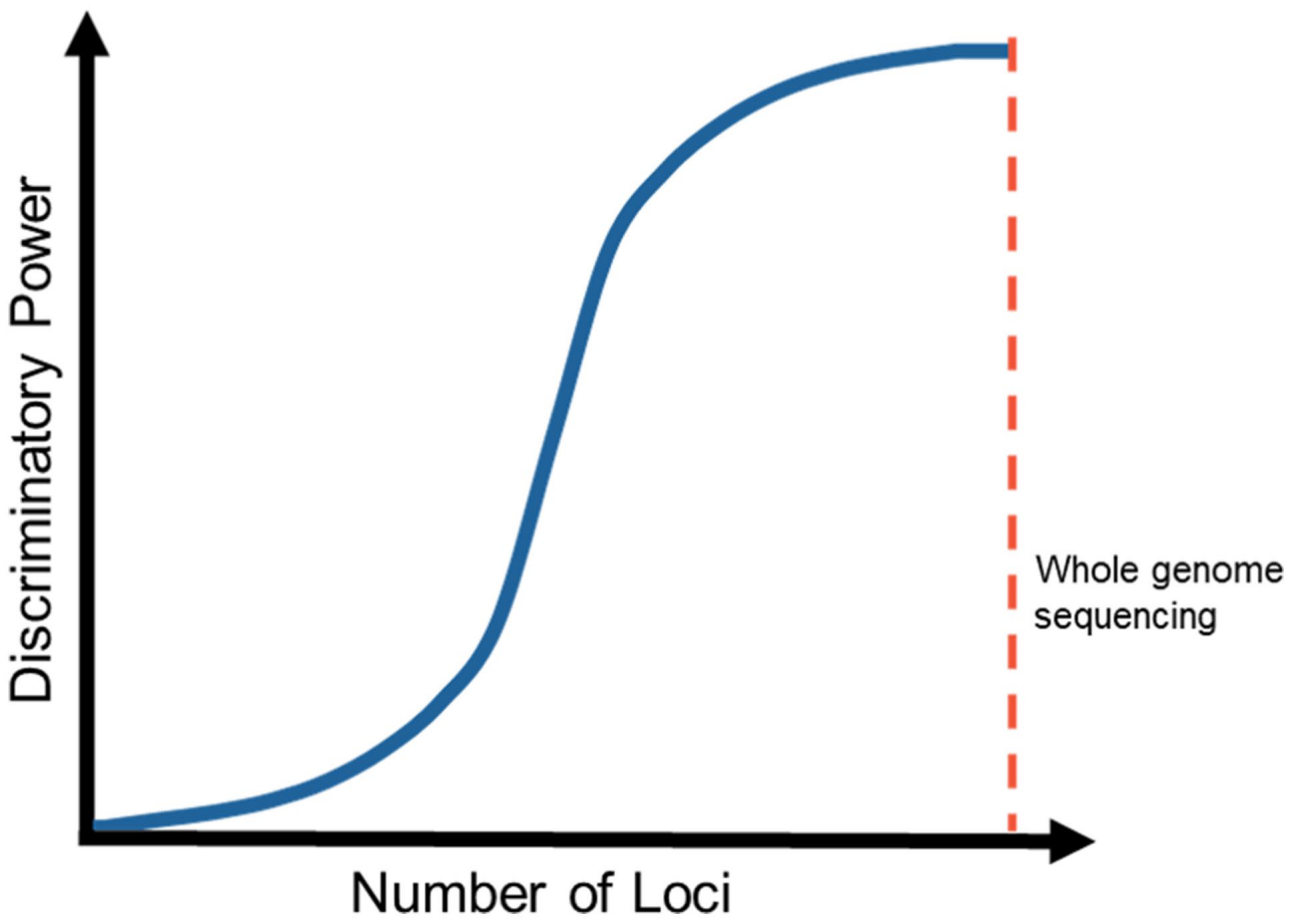
Potential cumulative increase in discriminatory power with the addition of more loci or whole genomes being used for identification.

It is predicted that with the implementation of a reliable and accurate whole-genome reference database, the accuracy of fungal identification will dramatically improve (Figure 5). The principle is demonstrated through the developing taxonomy of the *Fusarium solani* species complex. A previous study examined four loci of these species and proposed the change in nomenclature of the *F. solani* species complex to be placed in the *Neocosmospora* genus (Sandoval-Denis et al., 2019). However, a recent study has analyzed 19 loci and has maintained that the members of the *F. solani* species complex should remain in the genus *Fusarium* (Geiser et al., 2020; O'Donnell et al., 2020). As such, the increased loci analyzed generated more data and provided more insight into the taxonomy of the *F. solani* species complex and if whole-genome data were available, the most accurate taxonomic information would be revealed, which if applied to all (pathogenic) fungal species would greatly increase the discriminatory power of long-read sequencing in clinical diagnosis.

This highlights that global efforts are urgently needed to improve the relevant reference genome databases (FungiDB and GenBank) as well as the antifungal resistance database MARDy^7^ (Nash et al., 2018), to be able to open up the full potential of the metagenomic data generated by long-read sequencers.

### Bioinformatics

Currently, there is a wide variety of bioinformatics tools for the analysis of long-read sequences (Makalowski and Shabardina, 2020). Some such bioinformatics tools used for species identification in metagenomic studies are “What’s In My Pot” (Juul et al., 2015), SUPRI real-time (Gu et al., 2021), MetaMaps (Dilthey et al., 2019), BugSeq (Fan et al., 2021), and NGSpeciesID (Vasiljevic et al., 2021).

For use in a clinical setting, a simple bioinformatics workflow would be necessary for practical implementation into routine diagnosis as it would be best to avoid requiring highly specialized personnel for analysis. A study comparing the ability of different bioinformatics tools to identify a wide array of pathogens from long-read sequencing dataset would be necessary to determine the most suitable bioinformatics tools.

Data interpretation is a major issue in metagenomics and metabarcoding analysis. Both methods generate large amounts of data and for the purpose of clinical diagnosis, the interpretation can be complex. When samples are taken from sterile sites, the expected sequencing results would consist of predominantly *Homo sapiens* reads and reads assigned the causative pathogen. However, if any contamination was to occur and reads were assigned to multiple microbes, this would complicate identification of the causative pathogen. This issue is magnified when patient samples are taken from non-sterile sites. Sequencing results will have reads assigned to multiple microbes and although it would be intuitive to assume the microbe with the highest abundance would be the causative pathogen, this is not always the case as has been observed in Irinyi et al. (2020). To overcome this, the application of machine-leaning algorithms helping to distinguish true pathogens from common colonizers or environmental contaminants may one of the ways forward. The large proportion of *H. sapiens* reads also complicates data interpretation as percentages of reads assigned to microorganisms is comparatively small. However, if bioinformatics tools were designed to remove host reads, microorganism abundance can be more easily compared and may further illuminate the causative pathogen.

## The Future of Long-Read Sequencing and Fungal Diagnosis

The introduction of long-read sequencers from PacBio and ONT has allowed sequencing of genetic material in a way previously not possible. The first applications of long-read sequencing to identify agents of infectious disease has demonstrated its potential in clinical diagnosis.

The current state of DNA extraction technologies, the lack of comprehensive reference databases, and simple bioinformation pipelines requires further development before long-read sequencers can be implemented in routine diagnosis. Additionally, the identification of multiple microbes within a clinical specimen may not directly lead to a specific diagnosis, but in conjunction with the patient’s presentation and further testing should seriously be considered as a way to achieve a fast and reliable diagnosis. Further advancements in the clinical diagnostic metagenomics workflow must be made to make its implementation in routine clinical diagnostic feasible. With the rapid decline of sequencing costs and the exponential improvement in sequencing capabilities, long-read sequencing is an important new technology which may eventually reduce the impact of pathogenic fungi on human health, by drastically reducing the turnaround time for diagnosis, supporting better treatment choices, reducing morbidity and mortality, and massively reduce associated healthcare costs, through its use in clinical diagnosis.

## Author Contributions

WM, LI, and BS conceived the review. WM coordinated and supervised the review. MH, LI, YH, BS, and WM wrote and corrected the manuscript. All authors contributed to the article and approved the submitted version.

## Funding

This study was supported by a National Health and Medical Research Council of Australia (NH&MRC) grant (no. APP1121936) to WM.

## Conflict of Interest

The authors declare that the research was conducted in the absence of any commercial or financial relationships that could be construed as a potential conflict of interest.

## Publisher’s Note

All claims expressed in this article are solely those of the authors and do not necessarily represent those of their affiliated organizations, or those of the publisher, the editors and the reviewers. Any product that may be evaluated in this article, or claim that may be made by its manufacturer, is not guaranteed or endorsed by the publisher.

## Footnotes

^1^ https://unite.ut.ee/

^2^ https://www.boldsystems.org/

^3^ https://www.ncbi.nlm.nih.gov/refseq/

^4^ https://its.mycologylab.org/

^5^ https://fungidb.org/

^6^ https://www.ncbi.nlm.nih.gov/genbank/

^7^ http://mardy.dide.ic.ac.uk/
